# Relationship Between Type 2 Diabetes and White Matter Hyperintensity: A Systematic Review

**DOI:** 10.3389/fendo.2020.595962

**Published:** 2020-12-21

**Authors:** Dan-Qiong Wang, Lei Wang, Miao-Miao Wei, Xiao-Shuang Xia, Xiao-Lin Tian, Xiao-Hong Cui, Xin Li

**Affiliations:** ^1^Department of General Medical, Shanxi Bethune Hospital Shanxi Academy of Medical Sciences, Taiyuan, China; ^2^Department of Neurology, The Second Hospital of Tianjin Medical University, Tianjin, China; ^3^Department of Psychiatry, Shanxi Bethune Hospital Shanxi Academy of Medical Sciences, Taiyuan, China

**Keywords:** type 2 diabetes mellitus, white matter hyperintensities, relationship, microscopic, cognitive function, microangiopathy and neuropathy

## Abstract

White matter (WM) disease is recognized as an important cause of cognitive decline and dementia. White matter lesions (WMLs) appear as white matter hyperintensities (WMH) on T2-weighted magnetic resonance imaging (MRI) scans of the brain. Previous studies have shown that type 2 diabetes (T2DM) is associated with WMH. In this review, we reviewed the literature on the relationship between T2DM and WMH in PubMed and Cochrane over the past five years and explored the possible links among the presence of T2DM, the course or complications of diabetes, and WMH. We found that: (1) Both from a macro- and micro-scopic point of view, most studies support the relationship of a larger WMH and a decrease in the integrity of WMH in T2DM; (2) From the relationship between brain structural changes and cognition in T2DM, the poor performance in memory, attention, and executive function tests associated with abnormal brain structure is consistent; (3) Diabetic microangiopathy or peripheral neuropathy may be associated with WMH, suggesting that the brain may be a target organ for T2DM microangiopathy; (4) Laboratory markers such as insulin resistance and fasting insulin levels were significantly associated with WMH. High HbA1c and high glucose variability were associated with WMH but not glycemic control.

## Introduction

In 1987, Hachinski et al. first used the low density of computed tomography to represent diffuse white matter changes, called leukoaraiosis (LA) or rare white matter (1). White matter hyperintensities (WMH) are defined as patchy areas of signal hyperintensity scattered in the deep or periventricular white matter evident on brain magnetic resonance imaging (MRI) T2-weighted or Fluid Attenuation Inversion Recovery (FLAIR) sequences (2). This high signal on MRI is caused by cerebral small vessel disease (CSVD), also known as white matter lesions (WML) (3–5). Therefore, different terms such as WMH, WML, white matter disease, and LA are considered synonyms (2). We use the term WMH in this review.

WMH prevalence was shown to increase with age (6). The prevalence of WMH is as high as 70% in Chinese population aged between 35 and 80 years (7), and nearly 90% in the European population aged between 60 and 90 years (8). In a meta-analysis study, WMH were reported to be linked to an increased risk of stroke, dementia, and death (9). Therefore, understanding the causes of WMH is essential for the prevention of cerebrovascular disease. Advanced age and hypertension are closely associated with WMH burden (10–12). The relationship between diabetes and the WMH burden remains controversial.

Type 2 diabetes mellitus (T2DM) prevalence has been increasing rapidly over the past few decades worldwide (13).Based on recent International Diabetes Federation (IDF) estimates, in 2017, there were 451 million adults (age: 18–99 years) with diabetes globally, a figure that is predicted to reach 693 million by 2045 (14, 15). T2DM is considered a risk factor for cerebrovascular disease (16). There is strong evidence that T2DM is associated with an increased risk of stroke (17, 18), dementia (19), depression, and cognitive impairment (20, 21). It is also thought to be related to CSVD, such as WMHs and brain atrophy (22–25).

In this review, we assessed the relationship between T2DM and WMH, and the impact of decreased white matter integrity on cognition in patients with T2DM. Del Bene et al. (26) investigated the correlation between T2DM and LA in 2015 and found that the relationship between LA and T2DM was more consistent than previously reported. However, due to the heterogeneity of the research methods, no clear conclusions were reached. Therefore, we searched the recent relevant literature and conducted a systematic review and comprehensive analysis again.

## Methods

We use the following term combinations: white matter lesion MRI diabet*, white matter hyperintensity white matter hyperintensities MRI diabet*, white matter change OR white matter changes AND MRI AND diabet*, and leukoaraiosis AND MRI AND diabet*. A literature search was conducted using PubMed and Cochrane.

Inclusion criteria: (1) Recently published English literature; (2) Definite imaging findings of T2DM or prediabetes and abnormal white matter structure; (3) White matter structural abnormalities measured by MRI; (4) Quantitative measurements of the association between T2DM or prediabetes and abnormal white matter structure were provided; (5) Cross-sectional, case-control or cohort epidemiological studies or Mendelian randomization studies were used.

Exclusion criteria: (1) Articles not focusing on the relationship between T2DM and white matter lesions; (2) The same literature retrieved by different search terms; (3) Articles on the relationship between type 1 diabetes mellitus and white matter lesions; (4) Duplicate publications or review articles of the same data set; (5) Articles dealing with animal research and genetic factors; (6) Review articles.

Quality assessment: The methodological quality of the cross-sectional studies included was assessed using an 11-item checklist that was recommended by the Agency for Healthcare Research and Quality (AHRQ). An item would be scored “0” if it was answered “NO” or “UNCLEAR”; if it was answered “YES”, then the item scored “1”. Article quality was assessed as follows: low quality = 0–3; moderate quality = 4–7; high quality = 8–11. The Newcastle–Ottawa Scale (NOS) was used to assess each of the cohort studies’ quality by two independent authors. The NOS consists of three parts: selection (0–4 points), comparability (0–2 points), and outcome assessment (0–3 points). NOS scores of six were assigned as high-quality studies.

We explored the possible links between the presence of T2DM, the duration or complications of diabetes, laboratory markers such as glycated hemoglobin concentration, insulin resistance, and WMH or its progression over time. In addition, we also considered the effect of the relationship between T2DM and WMH on cognitive function.

## Results

### Literature Information

**Figure 1:**
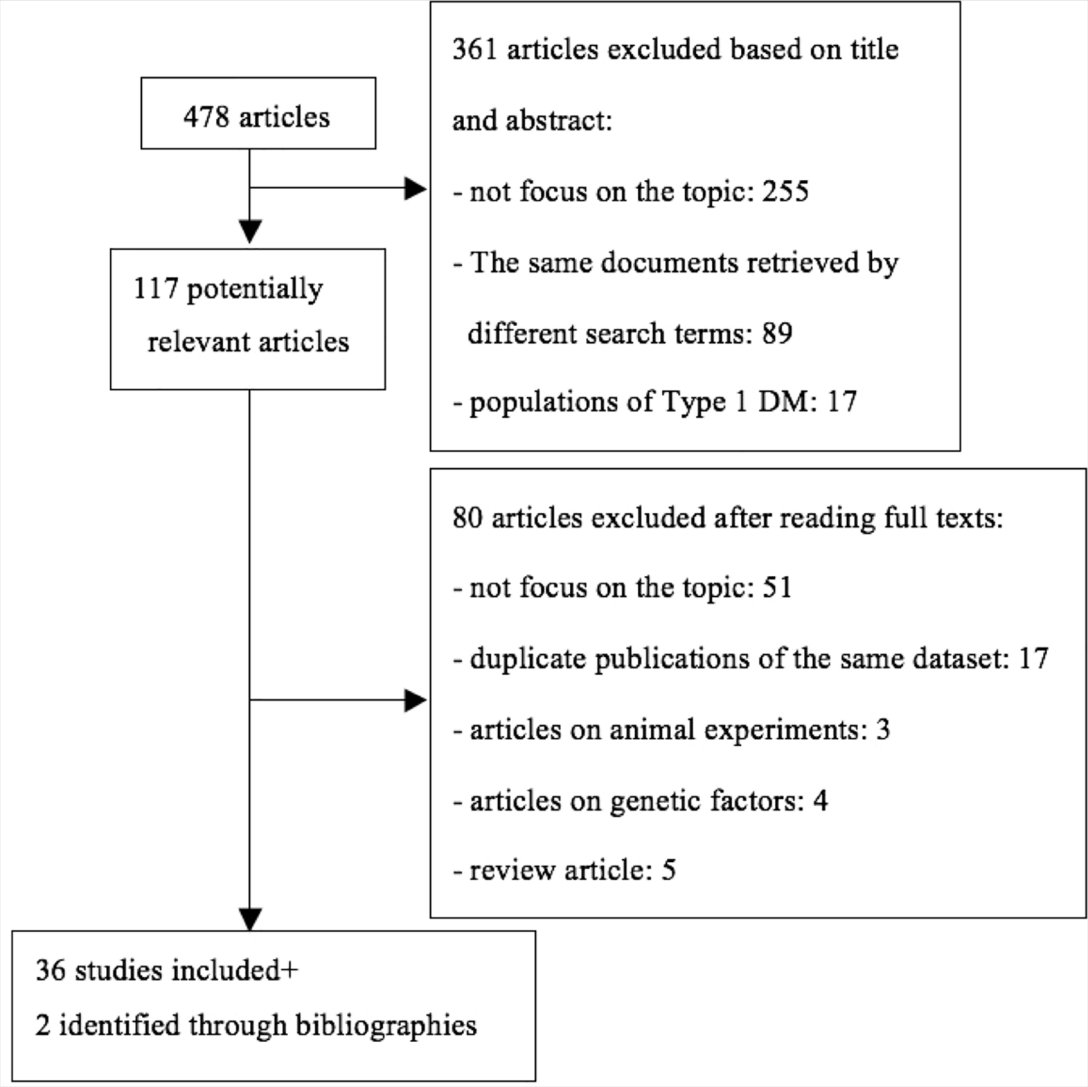
Summary of literature search.

A literature search was conducted using PubMed and Cochrane, and 478 articles published between January 2015 and December 2019 were retrieved. After title and abstract screening, 117 articles were retrieved, and 38 articles were finally included, including two obtained from the retrieved literature references (**Figure 1**).

In the included studies, WMH is evaluated by conventional MRI sequences. The volume was measured by visual rating scales, automatic or semi-automatic segmentation methods, or through unconventional MRI modalities, such as MRI-DTI or diffusion kurtosis imaging (DKI) methods. In each of the included studies, we recorded the study design, sample size, mean age, neuroimaging methods, study results, and correction parameters (**Table 1**).

**Table 1:** The study design, sample size, mean age, neuroimaging methods, study results, and correction parameters in the included studies.

Number	Study	Study Design	Subjects (n) total/type 2 DM	Mean age (years)	Imaging	WMH assessment method	Results	P-value	Adjustments
1	de Bresser et al. (27)	CS	114/60	71	MRI-FLAIR	WMH shape	a different shape (eccentricity) of punctuate deep WMH in T2DM	(Beta (95% CI): 0.40 (0.23↔0.58))	No
2	Lucatellia et al. (28)	CS	93/19	71 ± 9	MRI-FLAIR	WML volume and number	WMLV associated with diabetes WMLN associated with diabetes	P < 0.0001 P = 0.001	age, smoking status, gender, hypertension, hyperlipidemia, Coronary artery disease, Ischemic event
3	de Groot et al. (29)	CS	4532/406	63.8	MRI-DTI	FA	FA reduction (the association tracts and the forceps minor WM)in DM	Bonferroni correction	age,WMH
4	Ferik et al. (30)	L	136/66	54.5 ± 7.6	MRI-T1T2 FLAIR	DWMLs	DWMLs were more frequent in patients with polyneuropathy	p = 0.016	age, duration of DM
5	Groeneveld et al. (31)	CS	48(T2DM)/25(T2DM with MCI or early dementia	76.4 ± 5.0(T2DM with cognitive impairment) 76.5 ± 4.8(without cognitive impairment)	MRI-DTI	WMH volume FA MD	No association	NS	NO
6	Marseglia et al. (24)	CS L	2746/1189 (Prediabetes and diabetes)	74.1 ± 9.5(Diabetes) 74.6 ± 10.4(Prediabetes) 71.3 ± 10.0(Diabetesfree)	MRI-FLAIR	WMHV	DM associated with larger WMHV diabetes was associated with a faster increase in WMHV	p = 0.004 p = 0.023	sex, education, SES, BMI, hypertension, heart disease
7	Raffield et al. (32)	CS	784/682	65.89	MRI-T1 DTI	WMLV、FA、MD	increased WMLV inT2DM reduced white matter FA inT2DM increased white matter MD inT2DM	p=0.008 p ≤ 0.001 p ≤ 0.031	age, scanner, sex, history of cardiovascular disease, smoking, statin use, educational attainment, and blood pressure medication use
8	Freedman et al. (33)	CS	584/584	60.1 ± 7.9	MRI-T1T2FLAIR	WMLV	higher UACR with higher WMLV higher eGFR with lower WMLV	P=0.05 P<0.001	age, sex, level of education, body mass index, hemoglobin A1c concentration, hypertension, diabetes duration, smoking status, history of cardiovascular disease, eGFR, UACR
9.1	Hughes et al. (34)	CS	584/584	60.1 ± 7.9	MRI-T1T2FLAIR	WMLV	Higher WMV with poorer3MSE/MMSE performance higher Stroop interference	p = 3.6 ×10^−3^ p = 7.5 × 10^−3^	age, sex, BMI, hemoglobin A1c, hypertension, smoking, CVD, diabetes duration, LDL-cholesterol of education, MRI scanner, total intracranial volume
9.2	Whitlow et al. (35)	CS	263/263	60.4	MRI-T1FLAIR	WMLV	Higher WMLV was associated with poorer performance	P<0.05	age, sex, body mass index, HbA1c, level of education
10	Liang et al. (36)	CS	60/30(Prediabetes)	55.03 ± 6.66(Prediabetes)/52.80 ± 7.53(Control)	MRI-DTI	FA	the FA(bCC SLF.R SLF.L) decreased in the prediabetic group	p=0.035p=0.047 p=0.040	NO
11	Vergoossen et al. (37)	CS	2219/858(prediabetes and diabetes)	59 ± 8	MRI WM tractography	node degree	lower local efficiency with prediabetes lower clustering coefficient with prediabetes higher communicability withT2DM	P=0.033 P=0.049 P=0.008	age, sex, education, average node degree, MRI date
12	van Agtmaal et al. (38)	CS	2,228/855(prediabetes and diabetes)	59.2 ± 8.2	MRI-T1T2 FLAIR	WMHV	larger WMHVs(dWMHs and pWMHs) with prediabetes and T2DM	Ptrend <0.001	age, sex, intracranial volume, time between baseline and MRI measurements, BMI, smoking status, total–to–HDL cholesterol ratio, office systolic blood pressure, estimated glomerular filtration rate, educational level
13	Hsu et al. (39)	CS	1,204(diabetes)	62.8 ± 10.3	MRI	WMHV	WMLV higher in African Americans than European Americans	p=0.001	age, sex, diabetes duration, BMI, HbA1c, total intracranial volume, scanner, statins, CVD, smoking, hypertension
14	Weinstein et al. (40)	CS	1,597/124(diabetes andFBG)	40.3 ± 8.5	MRI-FLAIR DTI	WMHV FA	larger WMHV with diabetes lower FA with diabetes and greater FBG	p=0.015 P<0.05	age, sex, time between examination 1 and MRI
15	Ben Assayag et al. (41)	L	392/118	67.4 ± 9.7	MRI- DTI	FA MD	decreased NAWM FA with T2DM increased NAWM MD with T2DM	p=0.006 p=0.003	NO
16	Lin et al. (42)	CS	4,683/1102	64.3 ± 9.5	MRI-FLAIR	LA	LA early onset with diabetes LA late progression with diabetes	P<0.001 P<0.05	NO
17	Sanahuja et al. (43)	CS	312(153DR)	57(Diabetes without DR) 61(Diabetes with DR)	MRI-T2 FLAIR	WMLs	higher WML in patients with DR	p=0.04	NO
18	Hjortebjerg et al. (44)	CS	198/99	58	MRI-T2	WMLs	IGFBP-3 levels decreased with WMLs (Breteler score 0–2)	P < 0.05	age, sex, BMI, diabetes, triglycerides, fasting insulin, 24-h AMBP, 24 h heart rate, use of statins, antihypertensives
19	Espeland et al. (45)	L	319/164 (ILI)	45-76	MRI-T1 T2	WMHV	lower WMHV with lifestyle intervention participants	P = 0.02	intracranial volume, age, clinical site
20	Sudre et al. (46)	CS	469/178	71.5	MRI-T1 FLAIR	WMHV	Diabetes mellitus was not associated with higher WMHV	NS	NO
21	de Havenon et al. (47)	L	502/502	62.7 ± 5.7	MRI	progression of WMHV	A1c was not associated with WMH progression	NS	NO
22	Hawkins et al. (48)	CS	67/9	55.8	MRI-FLAIR	WMHV	Insulin predicted WMH	p < 0.001	NO
23	Sundar et al. (49)	CS	58	60	MRI	PVWMHs DWMHs	No association	NS	NO
24	Rist et al. (25)	L	345/50	74.4	MRI-T2 FLAIR	WMHV	No association	NS	age, sex, parent study, Physical activity, Smoking, Alcohol consumption, History of myocardial infarction,History of revascularization, History of cancer,History of hypertension, Hypertension treatment, History of high cholesterol, Statin use, History of TIA, History of angina, Hormone use, BMI
25	Tamura et al. (50)	CS	178/178	>65	MRI-T2 FLAIR	PVH DWMH	no association between HbA1c and WMH/IC GA/HbA1c associated with WMH/IC	NS p = 0.025	BMI, body mass index; BP, blood pressure
26	Power et al. (51)	L	1,851	75.3	MRI-DTI	FA MD	elevated glucose in midlife with worse late-life WM microstructural (MD) integrity	P = 0.0002	age, gender, education, race/center, BMI, the square of BMI, smoking status, APOE E4, the other vascular risk factors, antihypertensive medication use, antidiabetic medication use, lipid-lowering medications
27	Schneider et al. (52)	CS	1,713/1113	75.0(No diabetes)75.2 (Prediabetes)75.4(Diabetes HbA1c<7.0%) 75.1(HbA1c ≥7.0%)	MRI-T2 FLAIR	WMH	diabetes and HbA1c ≥7.0% with increased WMH	P = 0.016	age, sex, race/field center, education, smoking status, hypertension, cardiovascular disease, APOE e4 genotype, total intracranial volume
28	Liu et al. (53)	MR	8,429/253	62	MRI- FLAIR DTI	WMHV FA	no association lower FA was associated with T2DM	NS P=0.004	genotyping batch, age, sex, body mass index, blood pressure, ancestry-informative principal components
29	Sonoda et al. (54)	CS	143/143	71	MRI	PVH DSWMH	no association between PVH DSWMH and T2D eGFR	NS	age, sex, apolipoprotein E4 isoform, prior cardiovascular disease, duration of diabetes mellitus, HbA1c, hypertension, dyslipidemia, log-transformed urine albumin to creatinine ratio, education, log-transformed serum vitamin B12, log-transformed serum folate levels
30	Shen et al. (55)	CS	72/36	57.61 ± 6.21(T2DM) 56.19 ± 6.84(control)	MRI-DIR	WMHs	More WMHs in the diabetic group	p = 0.0015	NO
31	Reitz et al. (56)	CS	618/422	80.0	MRI	WMHV	higher levels of HbA1c were associated with increased WMHV	P<0.05	intracranial volume, age, sex, education, ethnic group, APOE
32	Nouwen et al. (57)	CS	53/14	16.1 ± 1.5(T2DM) 14.9 ± 2.00(Obese) 16.4 ± 1.7(Controls)	MRI-TBSS	FA	FA was negatively correlated HOMA-IR	p < 0.0001	age, BMI, HbA1c
33	Rofey et al. (58)	CS	25/15	18(T2DM) 15.4(Obese) 14.5(Control)	MRI-DTI	FA	lower FA(right hemisphere) with T2DM	p = 0.0023	NO
34	Xiong et al. (59)	CS	58/30	60.63 ± 6.01(T2DM) 58.54 ± 6.22(HC)	MRI-DKI	MK	decreased MK with T2DM	P<0.05	NO
35	Xiong et al. (60)	CS	68/42	59.05± 6.22(DM-NC) 62.75 ± 5.93(DM-MCI) 59.88 ± 6.17(Controls)	MRI-TBSS	FA MD	decreased FA(CT.R and CP.R) and increased MD(RIC.R and EC.R) with T2DM	p=0.003p=0.002 p=0.021p=0.009	NO
36	Zhuo et al. (61)	CS	66/40	41.7 ± 9.5(T2DM-NC) 47.3 ± 8.2 (T2DM-C) 42.5 ± 10.4(HCs)	MRI-TBSS	FA MD	no association in the FA values between the HCs and T2DM-NC decreased FA values and increased MD in the T2DM-C	NS P<0.05	NO
37	Gao et al. (62)	CS	116/76	65.97 ± 7.81(T2DM-aMCI) 65.35 ± 7.77(T2DM-NC) 66.48 ± 7.46(HC)	MRI-DTI	FA	no association in the FA values between T2DM-NC patients and HC decreased FA values in T2DM-aMCI	NS p<0.05	age, sex, education, BMI, hypertension, hyperlipidemia, and cerebrovascular disease
38	Espeland et al. (42)	L	319/319	69	MRI	WMHV	Women had a greater mean than men in summed WMH volumes	(95% CI): [0.00002, 2.78]	age, systolic and diastolic blood pressures

CS, cross-sectional; DKI, diffusion kurtosis imaging; DM, diabetes mellitus; DTI, diffusion tensor imaging; DM-NC,DM with normal cognition; DM-MCI,DM with mild cognitive impairment; FA, anisotropy fraction; FPG, fasting plasma glucose; HbA1c, glycated hemoglobin; L, longitudinal; LA, leukoaraiosis; MD, mean diffusivity; MRI, magnetic resonance imaging; MTI, magnetization transfer imaging; NS, not significant; WMHs, white matter hyperintensities; NAWM, normal-appearing white matter; FLAIR, Fluid attenuated inversion recovery; WML, white matter lesion; DWMLs, deep white matter lesions; PVWMHs, Periventricular white matter hyperintensities; DWMHs, Deep white matter hyperintensities; PVH, Periventricular hyperintensity; DIR, double inversion recovery; TBSS, Tract-Based Spatial Statistics; MK, mean kurtosis; HC, healthy controls; MR, Mendelian Randomization; GA/HbA1c, Glycoalbumin/Glycohemoglobin Ratio; IC, intracranial volume.

### Bias Risk Results for All Studies Included

All cross-sectional studies were assessed by AHRQ, eight of which were of high quality, and 30 of which were of moderate quality. All cohort studies were assessed by NOS, three of which were of high quality, and the rest were of medium quality.

### Macroscopic Analysis of the Relationship Between Type 2 Diabetes Mellitus and White Matter Hyperintensities

We define macro-analysis as the analysis of WMH volume in patients with T2DM by conventional MRI sequences (T1, T2, and FLAIR). Macroscopically, most studies have observed a larger WMH in T2DM. A recent cross-sectional studies (32, 40, 42, 55) using conventional MRI sequences and double inversion recovery (DIR) sequences have shown that patients with diabetes had more WMHs than the control group. In a prospective cohort study based on the elderly population (24), Marseglia et al. found that diabetes was cross-sectionally associated with larger WMH volume. Longitudinally, diabetes was associated with faster WMH accumulation. These findings highlight that diabetes may increase the accumulation of WMH markedly over time. Another middle-aged cohort study (38) showed that prediabetes and T2DM were significantly associated with larger volumes of WMHs compared with normal glucose metabolism (NGM). These results indicate that in middle-aged populations, structural brain abnormalities already occur in prediabetes. In a large community cross-sectional study (52), Schneider et al. found that ARIC-NCS participants with diabetes with HbA1c ≥ 7.0% have an increased burden of WMH, but those with prediabetes (HbA1c 5.7 to < 6.5%) and diabetes with HbA1c < 7.0% have WMH similar to those without diabetes.

Most studies on WMH have examined WMH volume, sometimes also addressing the number, location, and shape of WMH. Pierleone Lucatelli et al. (28) calculated WML volume and the number of lesions on FLAIR images using a semi-automated segmentation technique, and demonstrating a statistically significant correlation between T2DM and WMH in terms of lesion number and volume. Jeroen de Bresser et al. (27) artificially segmented the WMH of FLAIR images. With the marching cubes algorithm, a mesh is created to determine WMH shape and location features. In this study, T2DM had more non-punctuate WMH and a difference in shape (eccentricity) of punctuate deep WMH compared to controls, but patients with T2DM did not differ from controls on traditional WMH measures (total WMH volume).

However, it may be affected by the selection bias of imaging analysis methods and study populations. There are also studies showing no convincing link between T2DM and higher WMH. A multiethnic cohort of individuals surveyed (43) showed that T2DM was not convincingly associated with a higher lesion load, either when considering the population as a whole or in South-Asian or European subgroups separately. Sundar U’s study (49) also showed that among both diabetics and non-diabetics, no difference in periventricular white matter hyperintensities was found. Deep white matter hyperintensities were, conversely, seen only in the control group. Data from a prospective cohort study (25) also showed that a history of diabetes was associated with decreased white matter hyperintensity volume.

### Microscopic Analysis of the Relationship Between Type 2 Diabetes Mellitus and White Matter Hyperintensities

At present, diffusion tensor imaging (DTI) technology is mainly used to measure the microstructural changes of WM. We define micro-analysis as observed a decreased in the integrity of WMH in T2DM by DTI or DKI technology. The measures most often analyzed by DTI are fractional anisotropy (FA) and mean diffusivity (MD). Fractional anisotropy (FA) reflects the restriction of water molecule movement in all directions. Mean diffusivity (MD) is a scalar measure of how quickly water molecules diffuse overall (63, 64). The lower the FA, the higher the MD and the worse the microstructural integrity of WM. The Rotterdam Study (29) is a prospective cohort study based on the middle-aged and elderly population, which found significant changes in DTI measurements in T2DM. Persons with T2DM displayed loss of microstructural organization in the association tracts and the forceps minor reflected in a decrease in FA, and not related to age. A study of young and middle-aged adults (40) showed the strongest associations of diabetes and greater fasting blood glucose with reduced FA in WM. Tracts mostly implicated in those associations were the short association fibers, the inferior longitudinal fasciculus, the thalamic radiations, and the corpus callosum. The study found that hyperglycemia is associated with subtle brain injury even in young adults, indicating that brain injury is an early manifestation of impaired glucose metabolism. Two studies of adolescent T2DM and WMLs (57, 58) also found a reduction in FA in the T2DM group. Research data suggest that even adolescent T2DM is associated with differences in WM microstructure. A prediabetic study (36) also found WM microstructural damage in prediabetic patients. The predominant DTI abnormalities in prediabetic patients were mainly the reduction of FA values in the right part of the corpus callosum body (bCC), the right superior longitudinal fasciculus (SLF.R), and the left superior longitudinal fasciculus (SLF.L), suggesting that these areas may be the sites of the first lesion. On the other hand, the differences in MD, AD, and relative anisotropy (RD) between the two groups were not statistically significant in this study, suggesting that FA may be a more sensitive indicator of DTI parameter values. A European Mendelian randomization study (53) also found a significant association between T2DM and FA. Surprisingly, T2DM was not significantly associated with increased MD. It has been suggested that the FA decrease may be modulated more directly by myelin alterations, whereas MD is more sensitive to cellularity, edema, and necrosis (62). These results might, therefore, point to demyelination being an important factor in T2DM patients.

Several studies (32, 41, 60) have also found that T2DM status is simultaneously associated with reduced FA and increased MD. A longitudinal cohort study (51), the ARIC study quantified the relationship between vascular risk factors in midlife and later life and WM microstructural integrity in later life. The results showed that elevated glucose in midlife is related to worse WM microstructural integrity (reduced FA and increased MD) in later life.

In the Maastricht Study, Laura W. Vergoossen et al. (37) used whole-brain white matter tractography to find that prediabetes, T2DM, and continuous measures of hyperglycemia are associated with fewer white matter connections and weaker organization of white matter networks.

However, there are also some studies showing no convincing association between T2DM and poor WM microstructural integrity. Zhuo et al. (61) found that there was no significant difference in the FA values between the healthy controls (HCs) and T2DM patients without peripheral microvascular complications (T2DM-NC groups). Gao also (52) showed the T2DM-NC patients and HC individuals did not reveal any significant differences in WM integrity. However, the integrity of multiple WM tracts was impaired in patients with T2DM-aMCI (T2DM with amnesic mild cognitive impairment) compared with HC and T2DM-NC.

The observation of WM microstructures mostly uses DTI technology. DKI was developed to quantify the non-Gaussian property (65). Xiong et al. (59) used DKI to assess WM structure in patients with T2DM. They found T2DM patients had abnormalities, reflected by decreased FA and mean kurtosis (MK), in a variety of white matter regions, as well as in the thalamus and caudate. MK reductions occurred across a more extensive area than DTI metrics, such as in the pontine crossing tract, internal capsule, superior longitudinal fasciculus, corpus callosum, and gray matter structure. It indicated that DKI could provide more changes in the WM microstructure than DTI.

### Type 2 Diabetes Mellitus Affects Cognitive Function Through White Matter Hyperintensities

Although there is evidence that T2DM is independently associated with cognitive decline and WMH is associated with cognitive deterioration (50), whether T2DM affects cognition through WMH remains controversial. Two prospective cohort studies (24, 41) examined the relationship between T2DM and WMH and cognition at a longitudinal level. Anna Marseglia et al. (24) followed 455 people in the SNAC-K cohort for three or six years and found that T2DM increased the accumulation of WMH. Prediabetes and T2DM were observed to be independently associated with an accelerated decline in cognitive ability. Ben Assayag et al. (50) followed patients with first acute ischemic strokes or transient ischemic attacks (TABASCO cohort) for two years. They found that impaired baseline WM integrity in T2DM was independently associated with worse cognitive test performances two years later. In patients with T2DM, they observed impaired memory, executive function, and attention scores. This could be explained by the sensitivity of executive functioning and processing speed to delicate diffuse vascular-related deterioration in WM integrity. Weinstein’s findings (40) suggest that young adults and middle-aged persons with diabetes perform worse on tasks of verbal and visual memory, visual perception and attention, and have more severe white matter microstructural damage (decreased FA) compared to persons without diabetes. Studies of multiethnic groups (34, 35, 39) have found that T2DM is associated with brain atrophy and WML load, which in turn is associated with poorer cognitive performance. The current analysis of Caucasian and African-American groups in the United States with T2DM shows that there is consistent evidence of a correlation between poor performance on global executive function tests and abnormal brain structure. Two studies (60, 62) investigated WM microstructural changes by DTI analysis in patients with T2DM with mild cognitive impairment and those with normal cognition. The results showed that the episodic memory and attention function impairment in patients with T2DM has significant correlations with the integrity decline of white matter fibers in the right IFOF and right ILF. Memory changes in patients with T2DM are a gradual, continuous process that may not be adequately reflected by neuropsychological test scores but can be captured in DTI parameters. In a large cohort of older overweight or obese adults with T2DM, differences in brain volumes and white matter disease were apparent between women and men, but these did not account for a lower prevalence of cognitive impairment in women compared with men in this cohort. In the above studies, seven studies (24, 34, 35, 39, 40, 50, 62) adjusted for vascular risk factors such as hypertension, hyperlipidemia cardiovascular, and cerebrovascular diseases. This has implications that the observed relationship is true.

Although most studies have observed pathological vascular changes on MRI in patients with cognitive impairment and T2DM compared to patients without T2DM, other studies (31) have also observed that T2DM with cognitive impairment is mainly characterized by reduced gray matter volume and less disruption of white matter connectivity. These findings suggest that these vascular lesions such as WMH are not important determinants of the severity of cognitive impairment in patients with T2DM, and other etiologies need to be considered.

### Relationship Between Type 2 Diabetes Mellitus Microangiopathy, Neuropathy, and White Matter Hyperintensities

Four studies were found to correlate diabetic microangiopathy, peripheral neuropathy, and WMH (30, 33, 43, 61). Sanahuja et al. (43) observed 312 patients with T2DM. Of these, 153 patients (49%) had diabetic retinopathy (DR). It was found that the prevalence of WMH was higher in patients with DR, and the severity of WMH was associated with the presence of DR. Another study (33) examined 584 patients with T2DM and found that the larger the WMH volume, the higher the UACR and the lower the eGFR. Zhuo et al. (61) found that compared with T2DM-NC groups and HCs, the FA values in the corpus callosum, internal capsule, and other regions were significantly decreased in patients with T2DM-C. Sevgi Ferik (30) confirmed in a longitudinal study that there is a statistically significant association between polyneuropathy and Fazekas grade 1 or 2 DWMLs after adjusting for age, duration of T2DM and duration of polyneuropathy symptoms also found to be associated with DWMLs. These studies suggest that the brain may be a target organ for T2DM microangiopathy, similar to other classical target organs, such as the retina or kidney. Interestingly, Mika Sonoda et al. (54) conducted a cross-sectional study of 143 patients with T2DM who were not diagnosed with dementia or stroke. In multiple regression models adjusted for 12 potential confounders, eGFR was not significant in the presence of any type of WMH.

### Others

Four studies examined the association of duration of DM (32, 33, 46, 57) and HbA1c values (32, 47, 50, 56) with WMH, respectively. Christiane Reitz et al. (56) used HbA1c as a continuous variable to define the clinical type of dysglycemia. They explored the relationship between clinical types of dysglycemia and brain structure in older persons and found that higher levels of HbA1c were associated with an increased volume of WMH. The SABRE study (46) showed the duration of diabetes mellitus was only significantly associated with lesion load in the frontal periventricular regions for the European population. Yoshiaki Tamura et al. (50) found that WMH/IC was significantly associated with GA/HbA1c in a group of DM patients aged >65 years, suggesting glucose variability is the most influential factor, and the avoidance of glucose fluctuations may be a key strategy to prevent WMH. However, in the ACCORD study (47), Adam de Havenon et al. followed up for 40 months and then reported that the strict glycemic control group with HbA1c <6.0% was not associated with WMH progression. The results of Laura M. Raffield (32) were also not statistically significant, and those of Nouwen et al. (57) were consistent.

Insulin resistance (57), fasting insulin level (48), insulin-like growth factor (IGF) (44), and lifestyle intervention (45) were also found to be significantly associated with WMH. Arie Nouwen et al. (57) reported that an insulin resistance index (HOMA-IR) was the only independent predictor of FA, explaining 8.1% of the variance in FA in adolescents. Keith A. Hawkins et al. (48) showed that with shared variance controlled for, plasma insulin remained as significant predictors of WMH volume. The results of Rikke Hjortebjerg (44) showed that IGFBP-3 was negatively correlated with the severity of WMLs. In a longitudinal study (45), participants with T2DM were randomly assigned to receive ten years of lifestyle intervention. It was found that the mean (SE) WMH volume was 28% lower among lifestyle intervention participants compared with those receiving diabetes support and education. Corneal confocal microscopy (CCM) a surrogate marker of neurodegeneration has shown that the presence of WMH was associated with corneal nerve loss in patients with acute ischemic stroke after adjusting for age, diabetes, gender, dyslipidemia, and smoking (66).

## Discussion

In this review, we analyzed recently accumulated evidence to explore the link between the presence of T2DM and WMH or its progression over time, and also considered the impact of the relationship between T2DM and WMH on cognitive function. Our study shows that: (a) Both from a macro- and micro-scopic point of view most studies support this relationship of a larger WMH and a decreased in the integrity of WMH in T2DM. Using DTI or DKI technology to measure the microstructural changes of WM, most studies have observed a decrease in the integrity of WM in T2DM or prediabetes, especially in the FA values, mainly concentrated in the corpus callosum, combined tract and other parts; (b) From the relationship between brain structural changes and cognition in T2DM, the evidence that poor performance in memory, attention and executive function tests is associated with abnormal brain structure is consistent; (c) Diabetic microangiopathy or peripheral neuropathy may be associated with WMH, suggesting that the brain may be a target organ for T2DM microangiopathy; (d) Laboratory markers such as insulin resistance and fasting insulin levels were significantly associated with WMH, High HbA1c and high glucose variability are associated with WMH but not glycemic control.

The strengths of our study included: (1) We conducted a very thorough and extensive search on PubMed and Cochrane for all types of research in the past five years; (2) We comprehensively explored the relationship between T2DM and WMH in the elderly, middle-aged and young people, adolescents and different ethnic groups; (3) We analyzed the relationship between T2DM and the macrostructure and microstructural changes of WM from different perspectives.

There were some limitations to this research. (1) The results of this study are limited by the quality of these studies, especially the cross-sectional studies; (2) The heterogeneity of results may be due to differences in blood glucose measurement and the method of using and explaining the MRI; (3) The sample size of the selected studies was somewhat small and some articles did not provide the necessary information, so it is difficult for us to further analyze their heterogeneity; (4) Subgroup analysis was not possible due to the small number of studies; (5) We only included articles published in the English language. In future research, more advanced multiparametric MRI techniques should be used to study the structural changes of WM in T2DM and their effects on cognitive function, as well as potential pathophysiological mechanisms. In addition, future research should also pay more attention to the relationship between prediabetes or T2DM in adolescents, middle-aged and young adults, and abnormal WM structure, because abnormal WM structure may already occur in prediabetes or adolescent type 2 diabetes.

## Conclusion

Brain structural changes and associated cognitive decline in patients with T2DM can be detected by neuroimaging. Compared with structural magnetic resonance alone, advanced and novel functional magnetic resonance techniques are expected to reveal subtler brain changes. T2DM is directly associated with reduced WM integrity, which, in turn, leads to cognitive decline. Whether cognitive changes can be captured by multiparametric functional magnetic resonance imaging still requires high-quality, longitudinal, and long-term research to improve our understanding of this connection and cognition.

## Data Availability Statement

The original contributions presented in the study are included in the article/supplementary materials; further inquiries can be directed to the corresponding author.

## Author Contributions

W-DQ and WL conceptualized and designed the research. W-DQ and W-MM acquired the data. X-XS analyzed and interpreted the data. T-XL performed the statistical analysis. W-DQ and C-HX wrote the manuscript. WL and LX critically revised the manuscript for intellectual content. All authors contributed to the article and approved the submitted version.

## Funding

Funding of the study was provided by the National Key Research and development Plan “New Strategy on Pathogenesis and Clinical Diagnosis and Treatment of Chronic Cerebral Small Vessels” (No. 2016YFC1300600).

## Conflict of Interest

The authors declare that the research was conducted in the absence of any commercial or financial relationships that could be construed as a potential conflict of interest.
